# Letter from Chiavari, Italy

**Published:** 1886-04

**Authors:** A. Lagorio

**Affiliations:** Chiavari, Italy


### FOREIGN CORRESPONDENCE.

TO THE EDITORS OF THE *Chicago Medical Journal and Examiner.*

*Messrs. Editors.*—In addition to the new remedies that have lately attracted our attention, we have another which is worthy of mention. It has been extensively studied in France and in Italy by Dr. Pensato, who again called attention to it in a recent number of the *Riforma Medica.* This remedy has been known as methylbenzoil, acetobenzoil, methylacetone, phenylmethylcarbonile (Friedel), phenylmethylketone. But Dujardin-Beaumetz considering its physiological properties and the chemical group to which it belongs, named it hypnone. It was discovered by Friedel in 1857, and it is the type of a numerous class of mixed acetones, which are derived from two organic acids, one belonging to the series of the fatty acids, the other to that of the aromatic acids, from which the name of aromatic acetones given to this group of chemical bodies is derived.

Acetophenone is a mixed acetone derived by mixing ordinary acetone with benzophenone, and has for its formula $CH_3CO, C_6H_5$.

It is obtained by distillation (dry) from a mixture of acetate and benzoate of calcium, and when oxidized it changes into benzoic acid and carbonic acid. At ordinary temperatures hypnone is liquid, without color and of a persistent odor. Limousin has shown that it is not soluble in water or glycerine, but dissolves in alcohol, ether, chloroform, benzine, oil of turpentine and in essential oils.

From the researches of Dujardin-Beaumetz and others, it is known that physiological and toxic properties of hypnone differ very much in different animals. If fifty centigrams are injected under the skin of a guinea pig the animal will soon fall into a comatose condition and die in four to six hours. The rabbit is also very sensible to the action of hypnone; two grams do not kill the animal, but produce an insensibility at the point of injection. The temperature falls from 39.6° C. to 38° C., the urine smells strongly of hypnone, the animal will then fall into absolute inertia, but will be well again in twenty-four hours.

Laborde, Grasset, Dujardin-Beaumetz and Bordet have shown that three grams injected into a dog will have no effect, but if one or two cubic centigrams of hypnone is given by the stomach, a hypnotic action will follow. The hypnotic action is much increased if, according to Grasset, the remedy is introduced by intratracheal injections. A profound and lasting sleep will result without any danger to the animal. Intravenous injections will produce death; before dying there will occur a sound sleep, analgesia and anæsthesia, diminution of the vascular tension, and an irregular and interrupted respiration. In seventeen monkeys no hypnotic effect could be obtained, but instead a hypothermic action, at times slight and at times of many degrees, was observed. From all this evidence it will be seen that the drug acts on the nervous elements by diminishing the excitability, it lowers the vascular tension, and in toxic doses modifies the composition of the blood.

When given to a sound man in a dose of twenty or thirty centigrams in capsules united with sweet almond oil, the action is as follows: after a certain time, half to one hour, a calm and sound sleep is produced; the individual awakens without the headache, etc., which frequently occurs after a sleep produced by chloral and paraldehyde. The blood does not show any alteration, and the respiration and circulation are normal. Dubois has noticed that when an individual is under the effect of hypnone, he is much more easily brought under the influence of chloroform; a fact of much importance in surgical practice.

The main therapeutical indication of hypnone is to relieve insomnia. It is only beneficial in the so-called nervous insomnia, or in that produced by cerebral excitement, or by alcoholism, in which cases its action is far superior to all other hypnotics At a dose of twenty or forty centigrams, it produces a calm and sound sleep. It must not be given to individuals suffering with any cardiac affection. Dujardin-Beaumetz believes that hypnone is equal to chloral and paraldehyde. Somewhat inferior to chloral, for it does not possess any analgesic action, but superior to both because it provokes a calm sleep without disturbing the organism.

The best way to administer it is in capsules, each containing ten centigrams of the remedy dissolved in sweet almond oil. Dose, two to five capsules at once or at intervals of two to four hours. A. LAGORIO.

CHIAVARI, Italy, February 24th, 1886.

